# Supplementary material for: Lipophilic nanocrystal prodrug-release defines the extended pharmacokinetic profiles of a year-long cabotegravir
Source: Nat Commun. 2021 Jun 8;12:3453. doi: 10.1038/s41467-021-23668-x (PMC8187380; doi:10.1038/s41467-021-23668-x)
Supplement: Supplementary file 1 — Supplementary Information [file 41467_2021_23668_MOESM1_ESM.pdf]

Supplementary Information

Lipophilic Nanocrystal Prodrug-Release Defines the Extended Pharmacokinetic Profiles of a Year-Long Cabotegravir

Nagsen Gautam[a], JoEllyn M. McMillan[b], Devendra Kumar[a], Aditya N. Bade[b], Qiaoyu Pan[a], Tanmay A. Kulkarni[a], Wenkuan Li[a], Brady Sillman[b], Nathan A Smith[b], Bhagya L. Dyavar Shetty[b], Adam Szlachetka[c], Benson J. Edagwa[b], Howard E. Gendelman[a, b,*], Yazen Alnouti[a,*].

[a]*Department of Pharmaceutical Sciences, University of Nebraska Medical Center, Omaha, NE 68198 USA*

[b]*Department of Pharmacology and Experimental Neuroscience, University of Nebraska Medical Center, Omaha, NE 68198 USA*

[c]*Nebraska Nanomedicine Production Plant, University of Nebraska Medical Center, Omaha, NE 68198 USA*

*** These authors jointly supervised this work**

Yazen Alnouti, Ph.D., Department of Pharmaceutical Sciences, University of Nebraska Medical Center, NE 68198-6025, phone 402-559-4631; fax 402-559-9543
email yalnouti@unmc.edu (for correspondence).

Howard E. Gendelman, M.D., Department of Pharmacology and Experimental Neuroscience, University of Nebraska Medical Center, Omaha, NE. 68198-5880; phone 402 559 8920; fax 402 559 3733; email hegendel@unmc.edu (for submission and correspondence)

Supplementary Figures

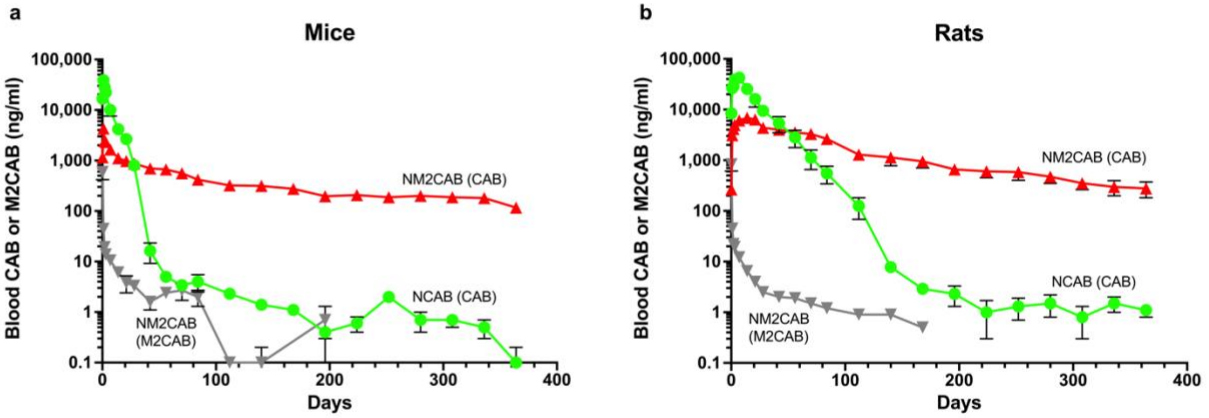

Supplementary Figure 1. **Blood CAB and M2CAB concentrations.** Blood concentration vs. time profiles for CAB and M2CAB over one year after a single IM injection (45 mg CAB equivalent/kg) of NCAB or NM2CAB in mice (**a**) and rats (**b**). Data are expressed as mean ± SEM (N = 6 animals per group per time point).

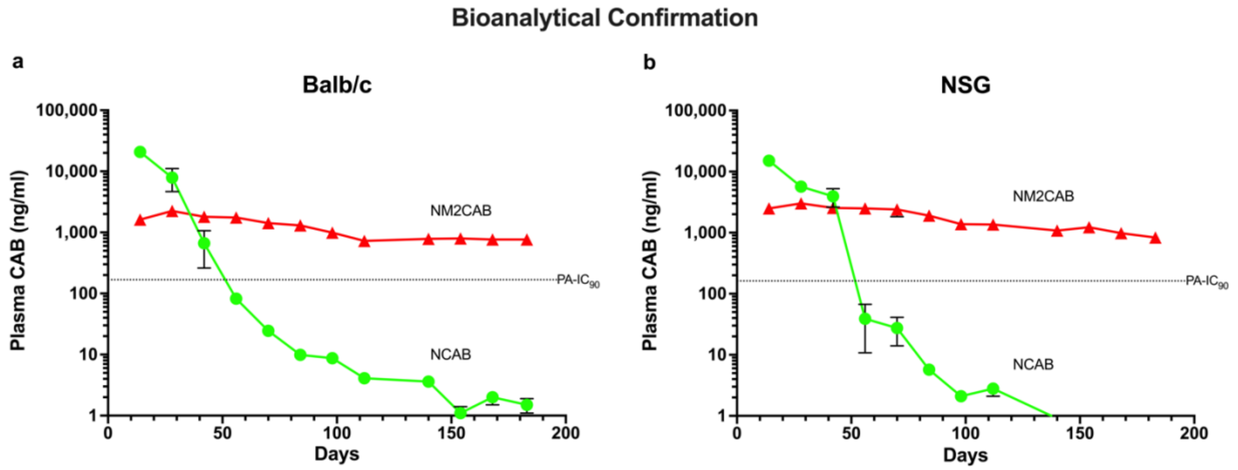

Supplementary Figure 2. **Bioanalytical confirmation of mouse plasma CAB concentrations.** Male Balb/c (**a**) or female NSG (**b**) mice were given a single intramuscular injection of 70 mg CAB equivalents/kg of NCAB or NM2CAB. Plasma was collected every other week for 6 months at which time the study was terminated. CAB concentrations were quantitated at UNMC by LC-MS/MS. The dotted line indicates the CAB protein-adjusted IC_{90} (PA-IC_{90}). Data are expressed as mean ± SEM (N varied from 2-6 animals per group per timepoint due to loss of animals by natural causes or inadequate plasma volume collected during the study period, with exact values for each time point provided in the source data [10.6084/m9.figshare.14498043]).

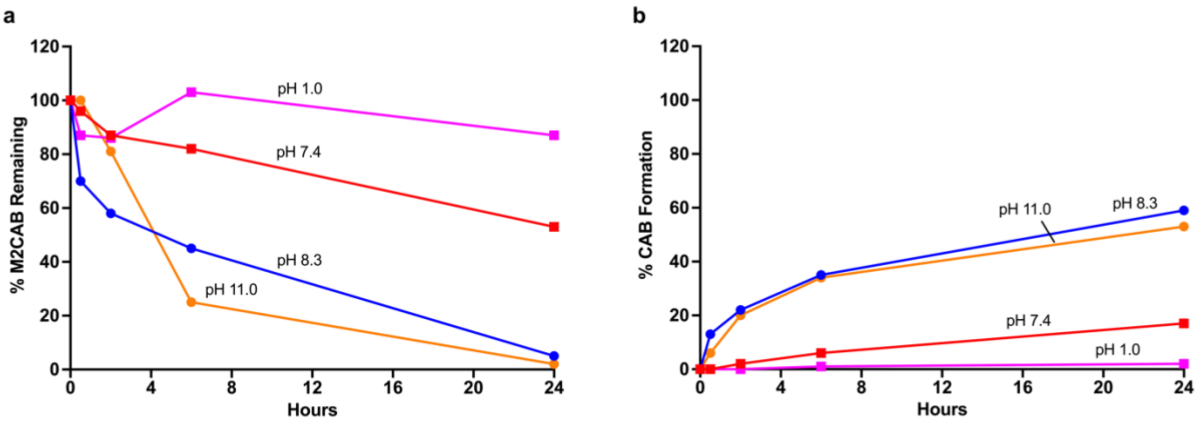

Supplementary Figure 3. **M2CAB pH stability.** Chemical stability profile of M2CAB at pH 1.0 (0.1 M HCl), pH 7.4 (PBS), pH 8.3 (heat inactivated plasma) and pH 11.0 (0.1 M NaOH) at 1 μM drug concentration. (**a**) Percent M2CAB remaining vs. time profile for M2CAB; (**b**) Percent CAB formation vs. time profile from M2CAB. Data are expressed as mean (N = 2 replicates per time point). Each experiment was repeated independently two times with equivalent results.

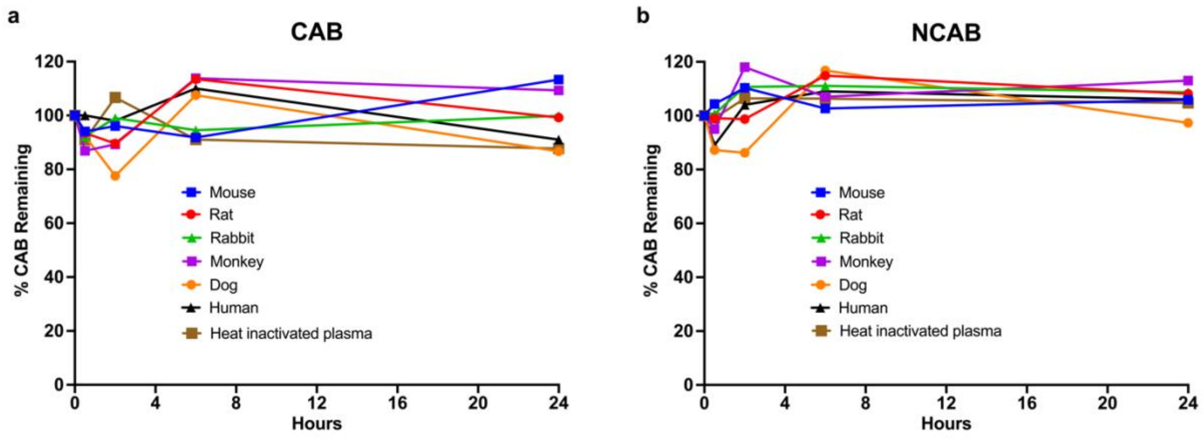

Supplementary Figure 4. **Plasma stability of M2CAB and NM2CAB.** Metabolic stability profiles of CAB (**a**) and NCAB (**b**) in six species (mouse, rat, rabbit, monkey, dog, and human) and heat-inactivated plasma at 1 μM concentration in blood. Data show CAB from CAB or NCAB treatment. Data are expressed as mean (N = 2 biological replicates per time point). Each experiment was repeated independently two times with equivalent results.

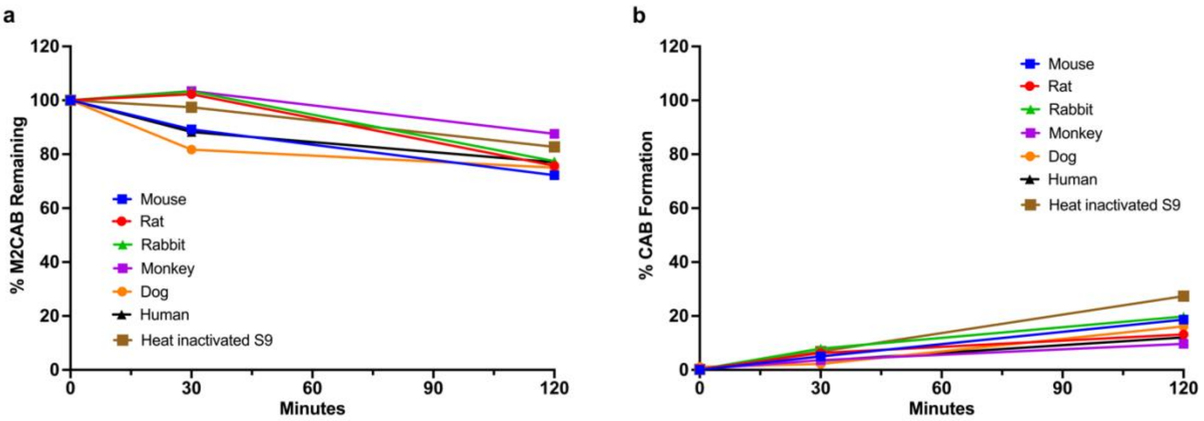

Supplementary Figure 5. **Liver S9 stability of M2CAB.** Metabolic stability profile of M2CAB at 1 μM concentration in liver S9 fraction from six species (mouse, rat, rabbit, monkey, dog, and human) and heat-inactivated liver S9 fraction. (**a**) Percent M2CAB remaining vs. time profile for M2CAB; (**b**) Percent CAB formation vs. time profile from M2CAB. Data are expressed as mean (N = 2 biological replicates per time point). Each experiment was repeated independently two times with equivalent results.

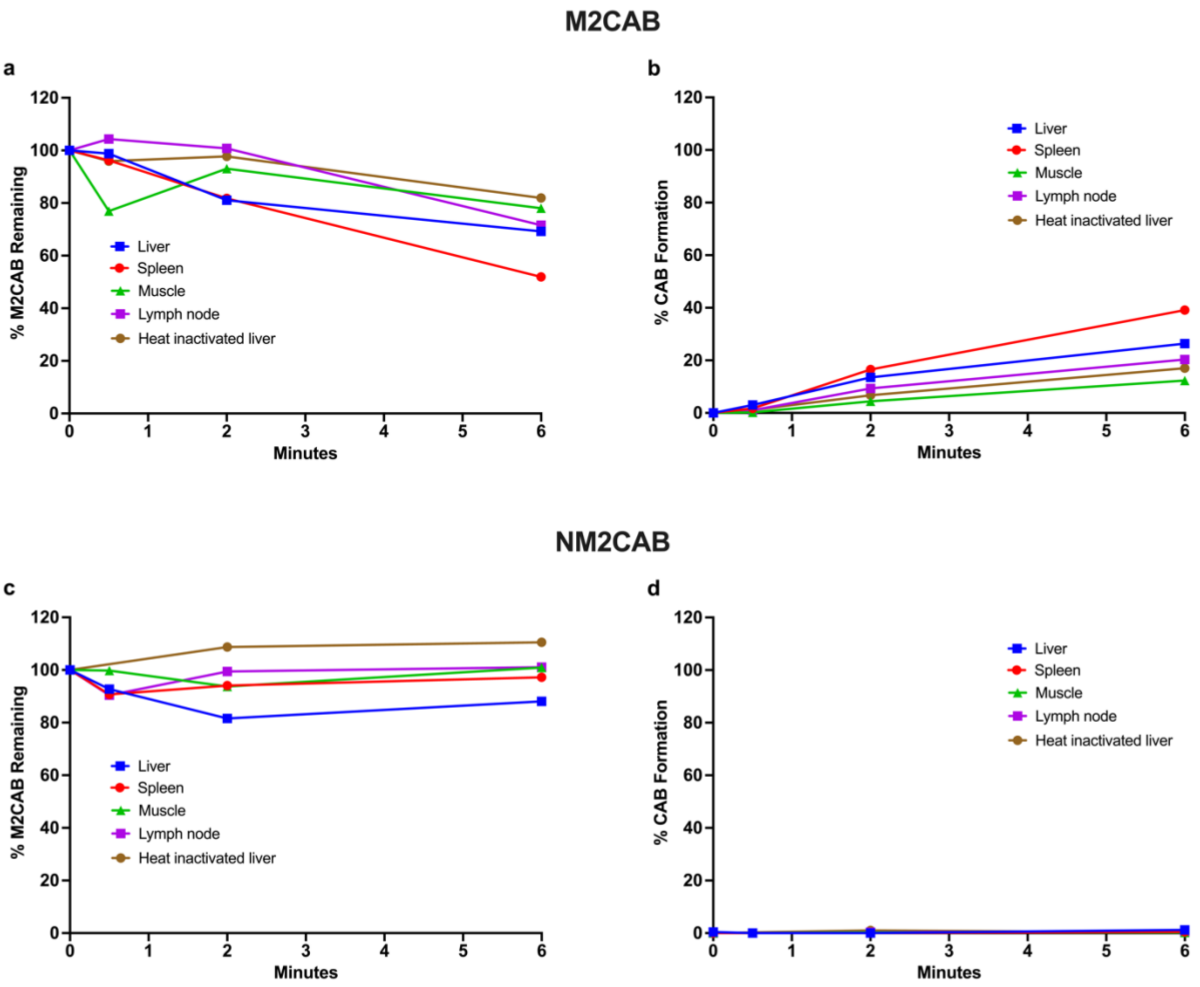

Supplementary Figure 6. **Tissue stability of M2CAB and NM2CAB.** Metabolic stability profiles of M2CAB, and NM2CAB at 1 μM concentration in rat tissues (liver, spleen, muscle, lymph node, and heat-inactivated liver). Data shown as M2CAB disappearance over time for (**a**) M2CAB and (**c**) NM2CAB and metabolite (CAB) formation from (**b**) M2CAB and (**d**) NM2CAB. Data are expressed as mean (N = 2 biological replicates per time point). Each experiment was repeated independently two times with equivalent results.

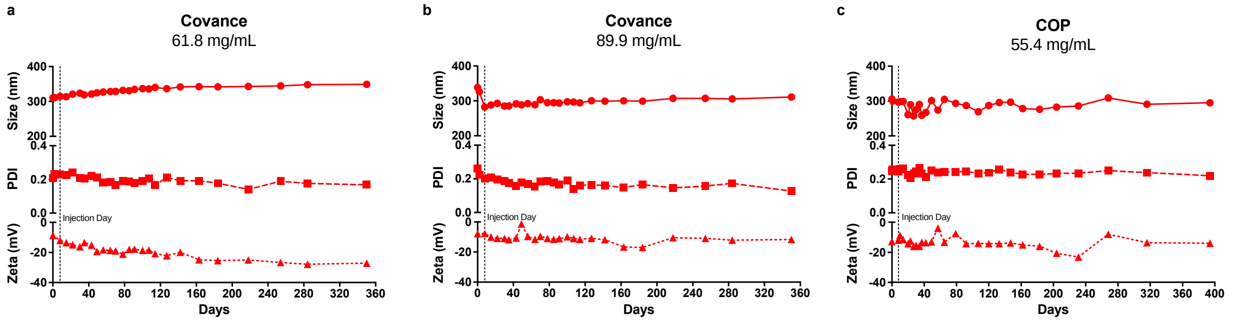

Supplementary Figure 7. **NM2CAB formulation stability.** Stability was tested on three separate NM2CAB formulations sitting on the benchtop at room temperature after manufacture for the Covance 45 mg CAB equivalents/kg (61.8 mg/ml; **a**), Covance 70 mg CAB equivalents/kg (89.9 mg/ml; **b**) and UNMC College of Pharmacy (COP; 55.4 mg/ml; **c**) PK studies. Formulation stability was measured by particle hydrodynamic diameter (size), polydispersity index (PDI), and zeta potential as determined by dynamic light scattering (DLS). Data are expressed as mean (N = 3 replicates per time point). Each experiment was repeated independently three times with equivalent results.

Supplementary Table 1**. Rat serum chemistry analysis.**

Serum Chemistry			Day 84				Day 365			
	Control		NCAB		NM2CAB		NCAB		NM2CAB	
	Mean	SEM	Mean	SEM	Mean	SEM	Mean	SEM	Mean	SEM
Albumin (g/dl)	4.7	0.3	4.7	0.2	5.0	0.3	4.6	0.2	4.7	0.1
Alkaline phosphatase (U/L)	13.3	2.3	17.0	4.5	11.0	3.2	17.7	2.9	14.3	0.7
Alanine aminotransferase (U/L)	74.0	15.9	41.7	2.4	44.0	2.9	82.7	15.6	60.3	5.8
Amylase (mg/dl)	850.0	68.4	626.0	61.9	527.7	70.1	573.0	45.1	690.7	46.2
Total bilirubin (mg/dl)	0.20	0.00	0.23	0.03	0.20	0.00	0.27	0.03	0.27	0.03
Blood Urea Nitrogen (mg/dl)	22.7	1.5	20.7	0.7	24.3	0.3	21.0	2.1	21.3	1.2
Creatinine (mg/dl)	0.50	0.00	0.37	0.12	0.60	0.00	0.37	0.09	0.57	0.03
Glucose (mg/dl)	127.0	10.5	150.0	6.5	178.0	9.2	146.0	8.5	175.0	11.1
Total protein (g/dl)	7.1	0.4	6.7	0.1	6.4	0.1	6.2	0.3	6.5	0.3
Globulin (g/dl)	2.4	0.7	1.4	0.1	1.5	0.2	1.7	0.2	1.9	0.3
Phosphorus (mg/dl)	5.2	0.6	7.0	0.5	6.4	0.7	5.8	0.4	5.5	0.4

N = 3 biological replicates
No statistical differences at $P = 0.05$ degrees of freedom = 4
Two-tailed Multiple *t*-test with Holm-Sidak multiple comparisons correction (Control vs. Treatment)
