# Supplementary material for: Lipophilic nanocrystal prodrug-release defines the extended pharmacokinetic profiles of a year-long cabotegravir
Source: Nat Commun. 2021 Jun 8;12:3453. doi: 10.1038/s41467-021-23668-x (PMC8187380; doi:10.1038/s41467-021-23668-x)
Supplement: Supplementary file 3 — Reporting Summary [file 41467_2021_23668_MOESM3_ESM.pdf]

nature research

Corresponding author(s): Yazen Alnouti, PhD, and Howard E. Gendelman, MD

Last updated by author(s): Apr 28, 2021

Reporting Summary

Nature Research wishes to improve the reproducibility of the work that we publish. This form provides structure for consistency and transparency in reporting. For further information on Nature Research policies, see our Editorial Policies and the Editorial Policy Checklist.

Statistics

For all statistical analyses, confirm that the following items are present in the figure legend, table legend, main text, or Methods section.

n/a	Confirmed	
☐	☒	The exact sample size (*n*) for each experimental group/condition, given as a discrete number and unit of measurement
☐	☒	A statement on whether measurements were taken from distinct samples or whether the same sample was measured repeatedly
☐	☒	The statistical test(s) used AND whether they are one- or two-sided<br>*Only common tests should be described solely by name; describe more complex techniques in the Methods section.*
☒	☐	A description of all covariates tested
☐	☒	A description of any assumptions or corrections, such as tests of normality and adjustment for multiple comparisons
☐	☒	A full description of the statistical parameters including central tendency (e.g. means) or other basic estimates (e.g. regression coefficient) AND variation (e.g. standard deviation) or associated estimates of uncertainty (e.g. confidence intervals)
☐	☒	For null hypothesis testing, the test statistic (e.g. *F*, *t*, *r*) with confidence intervals, effect sizes, degrees of freedom and *P* value noted<br>*Give P values as exact values whenever suitable.*
☒	☐	For Bayesian analysis, information on the choice of priors and Markov chain Monte Carlo settings
☒	☐	For hierarchical and complex designs, identification of the appropriate level for tests and full reporting of outcomes
☒	☐	Estimates of effect sizes (e.g. Cohen's *d*, Pearson's *r*), indicating how they were calculated

Our web collection on statistics for biologists contains articles on many of the points above.

Software and code

Policy information about availability of computer code

Data collection	MassLynx V4.1 software (Waters, Milford, MA)
Data analysis	MassLynx V4.1 software (Waters, Milford, MA); Microsoft Excel V16.45; GraphPad Prism V9.0.0.0; Non-compartmental PK analysis for plasma drug levels was performed using Phoenix WinNonlin-8.2 software (Certara, Princeton, NJ).

For manuscripts utilizing custom algorithms or software that are central to the research but not yet described in published literature, software must be made available to editors and reviewers. We strongly encourage code deposition in a community repository (e.g. GitHub). See the Nature Research guidelines for submitting code & software for further information.

Data

Policy information about availability of data

All manuscripts must include a data availability statement. This statement should provide the following information, where applicable:

- Accession codes, unique identifiers, or web links for publicly available datasets
- A list of figures that have associated raw data
- A description of any restrictions on data availability

The authors declare that the data supporting the findings of this study are available within the paper and its supplementary information files. Source data are provided with this paper.
DOI: 10.6084/m9.figshare.14496093, 10.6084/m9.figshare.14498025, 10.6084/m9.figshare.14498028, 10.6084/m9.figshare.14498034, 10.6084/m9.figshare.14498037, 10.6084/m9.figshare.14498043, 10.6084/m9.figshare.14498046, 10.6084/m9.figshare.14498049, 10.6084/m9.figshare.14498052, 10.6084/m9.figshare.14498055, 10.6084/m9.figshare.14498058

Field-specific reporting

Please select the one below that is the best fit for your research. If you are not sure, read the appropriate sections before making your selection.

☒ Life sciences ☐ Behavioural & social sciences ☐ Ecological, evolutionary & environmental sciences

For a reference copy of the document with all sections, see nature.com/documents/nr-reporting-summary-flat.pdf

Life sciences study design

All studies must disclose on these points even when the disclosure is negative.

Sample size	Sample sizes chosen were sufficient to determine significance in all assays, with reproducible statistically significant differences between experimental conditions. For comparing two groups for PK analysis, six animals/group will provide 80% power at the 0.05 level of significance to detect a difference of 2.0 standard deviations using a t-test. For animal studies, sample sizes were determined in order to provide statistical power while also meeting cost and ethical criteria for animal use.
Data exclusions	Exclusion criteria was predetermined. Extreme outliers beyond the 99% confidence interval of the mean and 3-fold greater than the SEM were excluded. No data were determined to fit this criteria, therefore no data points were excluded from the current reported values.
Replication	All attempts to reproduce the experimental findings were successful. For chemical synthesis, characterization, and formulation production, experiments were repeated independently a minimum of three times with similar results. For in vitro cellular assays, experiments were repeated independently a minimum three times with similar results. For prodrug hydrolysis studies, experiments were repeated independently two times with equivalent results. For animal studies, results of the year long study were validated in different strains of mice and species (mice, rats and rhesus macaques). Mouse studies were independently repeated three times (Fig. 1, 2, and Kulkarni et al. Fig. 4), rat experiments were conducted once with an N = 6 animals per group, and rhesus macaque studies were conducted once with an N = 4 animals.
Randomization	For all studies, samples/cells/animals were randomly allocated into experimental groups at the beginning of each study.
Blinding	Studies at UNMC were designed such that cell and PK data collection and analysis were blinded. However, investigators were not blinded in conducting experiments or sample collection, relying on an unbiased approach. Separate investigators conducted sample collection and data collection/analysis, providing additional support to the unbiased conduct of the studies. For animal studies, animals were randomized into treatment groups before study initiation. Personnel involved in drug administration, sample and data collection, and sample analysis at Covance were blinded as to treatments.

Reporting for specific materials, systems and methods

We require information from authors about some types of materials, experimental systems and methods used in many studies. Here, indicate whether each material, system or method listed is relevant to your study. If you are not sure if a list item applies to your research, read the appropriate section before selecting a response.

Materials & experimental systems

n/a	Involved in the study	
☒	☐	Antibodies
☐	☒	Eukaryotic cell lines
☒	☐	Palaeontology and archaeology
☐	☒	Animals and other organisms
☒	☐	Human research participants
☒	☐	Clinical data
☒	☐	Dual use research of concern

Methods

n/a	Involved in the study	
☒	☐	ChIP-seq
☒	☐	Flow cytometry
☒	☐	MRI-based neuroimaging

Eukaryotic cell lines

Policy information about cell lines

Cell line source(s)	Human peripheral blood monocytes
Authentication	Human peripheral blood monocytes were isolated by leukapheresis from HIV-1/2 and hepatitis B seronegative donors and purified by centrifugal elutriation from the University of Nebraska Medical Center (UNMC) Elutriation and Cell Separation Core according to a UNMC Institutional Review Board exempt protocol with informed consent. Monocytes were cultured for the first seven days in macrophage colony stimulating factor enriched media (MCSF, 1000 U/ml) to facilitate cell vitality and differentiation into macrophages.
Mycoplasma contamination	Cells obtained from elutriation were negative for mycoplasma
Commonly misidentified lines (See ICLAC register)	N/A

Animals and other organisms

Policy information about studies involving animals; ARRIVE guidelines recommended for reporting animal research

Laboratory animals	A) Mice: 1) NSG, Female, 6-8 weeks age and 2) BALB/cJ, Male, 6-8 weeks age;<br>B) Rats: Sprague Dawley, male, 8 weeks<br>C) Rhesus Macaques, Male, 5-6 years age (Kulkarni, T.A. et al. A year-long extended release nanoformulated cabotegravir prodrug. Nat Mater (2020) in press.)
Wild animals	The study did not involve wild animals.
Field-collected samples	The study did not involve samples collected from the field.
Ethics oversight	All experimental protocols involving the use of laboratory animals were approved by the UNMC or Covance Institutional Animal Care and Use Committees (IACUC) ensuring the ethical care and use of laboratory animals in experimental research. All animal studies were performed in compliance with institutional policies and NIH guidelines for laboratory animal housing and care in American Animal Association and Laboratory Animal Care (AAALAC) accredited facilities.

Note that full information on the approval of the study protocol must also be provided in the manuscript.
